# Adverse effects of long-term antipsychotic treatment in children and adolescents

**DOI:** 10.1192/j.eurpsy.2026.11780

**Published:** 2026-07-31

**Authors:** T. Gutierrez Higueras

**Affiliations:** Child and adoscent Psichiatry, Servicio Andaluz de Salud, Jaén, Spain

## Abstract

**Introduction:**

Antipsychotics are frequently prescribed in children and adolescents to treat various conditions such as schizophrenia, bipolar disorder, autism spectrum disorders, and severe behavioral disorders. These medications can be effective in reducing symptoms and improving overall functioning, but their long-term safety is largely unknown. Long-term treatment with antipsychotics can cause metabolic, neurological, endocrine, and cardiac disorders, affecting overall health and quality of life. Understanding these effects is key to optimizing long-term treatment strategies that balance benefits and risks.

**Objectives:**

To analyze the literature regarding adverse effects associated with long-term antipsychotic treatment in children and adolescents, identifying the most frequent ones and comparing different typical and atypical antipsychotics.

**Methods:**

A literature review was conducted in PubMed for studies published between 2015 and 2025. Systematic reviews and meta-analyses were prioritized. Studies with patients under 18 years of age, treatment duration ≥6 months, and outcomes were included.

**Results:**

Long-term antipsychotic treatment and cumulative dose were associated with the severity of adverse effects. The most common metabolic effects were weight gain and dyslipidemia. The most notable neurological effects were extrapyramidal symptoms and sedation. Endocrine disorders were most commonly reported as hyperprolactinemia, delayed puberty, and menstrual irregularities. The most significant cardiological effects were QT interval prolongation and arrhythmias.

**Conclusions:**

Long-term antipsychotic treatment in children and adolescents can cause significant adverse effects at the metabolic, neurological, endocrine, and cardiac levels. Comprehensive monitoring, periodic evaluation, and individualized treatment planning are necessary to balance therapeutic benefits with risks, considering minimal doses.

**Disclosure of Interest:**

None Declared

